# Gut Microbial Levodopa Metabolism and Constipation in Prodromal and Established Parkinson’s Disease

**DOI:** 10.3390/nu18213470

**Published:** 2026-10-22

**Authors:** Giovanni Albani, Paolo Capodaglio, Marica Giardini, Giuseppe Modaffari, Vasuki Ranjani Chellamuthu, Yin-Xia Chao, Anusha Jayaraman, Salvatore Albani, Eng-King Tan

**Affiliations:** 1 Division of Neurorehabilitation of Veruno Institute, Istituti Clinici Scientifici Maugeri IRCCS, 28013 Gattico-Veruno, Italy; giovanni.albani@icsmaugeri.it; 2 Research Laboratory in Biomechanics, Rehabilitation and Ergonomics, IRCCS, Istituto Auxologico Italiano, San Giuseppe Hospital, 28824 Piancavallo, Italy; p.capodaglio@auxologico.it (P.C.); g.modaffari@auxologico.it (G.M.); 3 Department of Biomedical, Surgical and Dental Sciences, University of Milan, 20122 Milan, Italy; 4 Translational Immunology Institute, SingHealth/Duke-NUS Academic Medical Centre, Singapore 169856, Singapore; vasuki.ranjani@visitor.nus.edu.sg (V.R.C.); salvo@duke-nus.edu.sg (S.A.); 5 National Neuroscience Institute, Singapore 308433, Singapore; chao.yinxia@singhealth.com.sg (Y.-X.C.); tan.eng.king@singhealth.com.sg (E.-K.T.); 6 Department of Neurology, Singapore General Hospital, Singapore 169608, Singapore; 7 Duke-National University of Singapore Medical School, Singapore 169857, Singapore

**Keywords:** enteric dopamine, Parkinson’s disease, constipation, non-motor symptoms

## Abstract

**Background:** Constipation can precede the motor diagnosis of Parkinson’s disease (PD), but its mechanisms are multifactorial. Gut bacteria can metabolize administered levodopa (LD), and enteric dopamine receptors participate in the regulation of motility. These observations motivate, but do not establish, a connection between microbial LD metabolism and constipation. **Evidence**: Bacterial tyrosine decarboxylase (TDC) converts LD to dopamine in biochemical and animal systems. Human studies associate fecal TDC abundance with some treatment and pharmacokinetic variables, but gene abundance does not measure enzyme activity. A longitudinal PD cohort found an inverse association between constipation severity and TDC abundance in one model, highlighting the need to consider medication effects and reverse causation. Microbiome alterations in risk-enriched populations do not demonstrate increased TDC activity before PD diagnosis. **Hypothesis:** In a subset of untreated individuals at increased risk of PD, sufficient luminal endogenous L-DOPA might permit microbial dopamine production that influences intestinal transit, provided that the resulting dopamine reaches relevant receptors at effective concentrations. Endogenous substrate availability, anatomical access, and the net balance of dopamine production and clearance are unresolved. After LD treatment begins, a larger exogenous substrate supply creates a distinct setting for drug–microbe interactions. **Conclusions:** The proposed continuum is conditional and requires biochemical and functional validation before human biomarker development. We propose substrate tracing, comparisons of TDC-positive and TDC-deficient bacteria, receptor-specific testing, and longitudinal gastrointestinal phenotyping. Current evidence does not justify TDC-directed dietary prevention of PD or clinical prediction from fecal TDC abundance alone.

## 1. Introduction and Scientific Scope

In everyday clinical practice, when treating a patient with Parkinson’s disease (PD), levodopa (LD) is prescribed primarily to improve motor function, while the fate of its intestinal substrate is less often considered. In this article, we review how dopamine is synthesized both within and outside the brain, and how diet and the disease itself can influence these two levels.

Recent research has identified bacterial enzymes that can metabolize the drug before or after its principal absorptive window. The final path of this narrative review leads to a hypothesis on whether these findings could also motivate a testable mechanism for constipation, as a non-motor symptom of prodromal and established PD.

## 2. Dopamine Synthesis and Intestinal Signaling

Dopamine is a catecholamine neurotransmitter that plays a pivotal role in motor control, motivation, reward processing, learning, and executive functions. Within the central nervous system, dopaminergic signaling regulates reinforcement learning and goal-directed behavior, whereas its dysfunction is a hallmark of several neurological and psychiatric disorders, particularly PD.

The principal sources of dopamine in the central nervous system are the dopaminergic neurons of the *substantia nigra pars compacta* and the ventral tegmental area. These nuclei give rise to the major dopaminergic pathways, including the nigrostriatal (motor control), mesolimbic (reward and motivation), and mesocortical (cognition and executive function) systems.

Dopamine synthesis requires distinct enzymatic steps: host tyrosine hydroxylase converts tyrosine to LD, and aromatic L-amino acid decarboxylase (AADC) converts LD to dopamine. Tyrosine hydroxylase activity is regulated and depends on tetrahydrobiopterin and iron; dietary precursor intake therefore cannot be translated directly into a proportional increase in dopamine synthesis [1]. Newly synthesized dopamine is then transported into synaptic vesicles by VMAT2 for storage and activity-dependent release.

**Figure 1:**
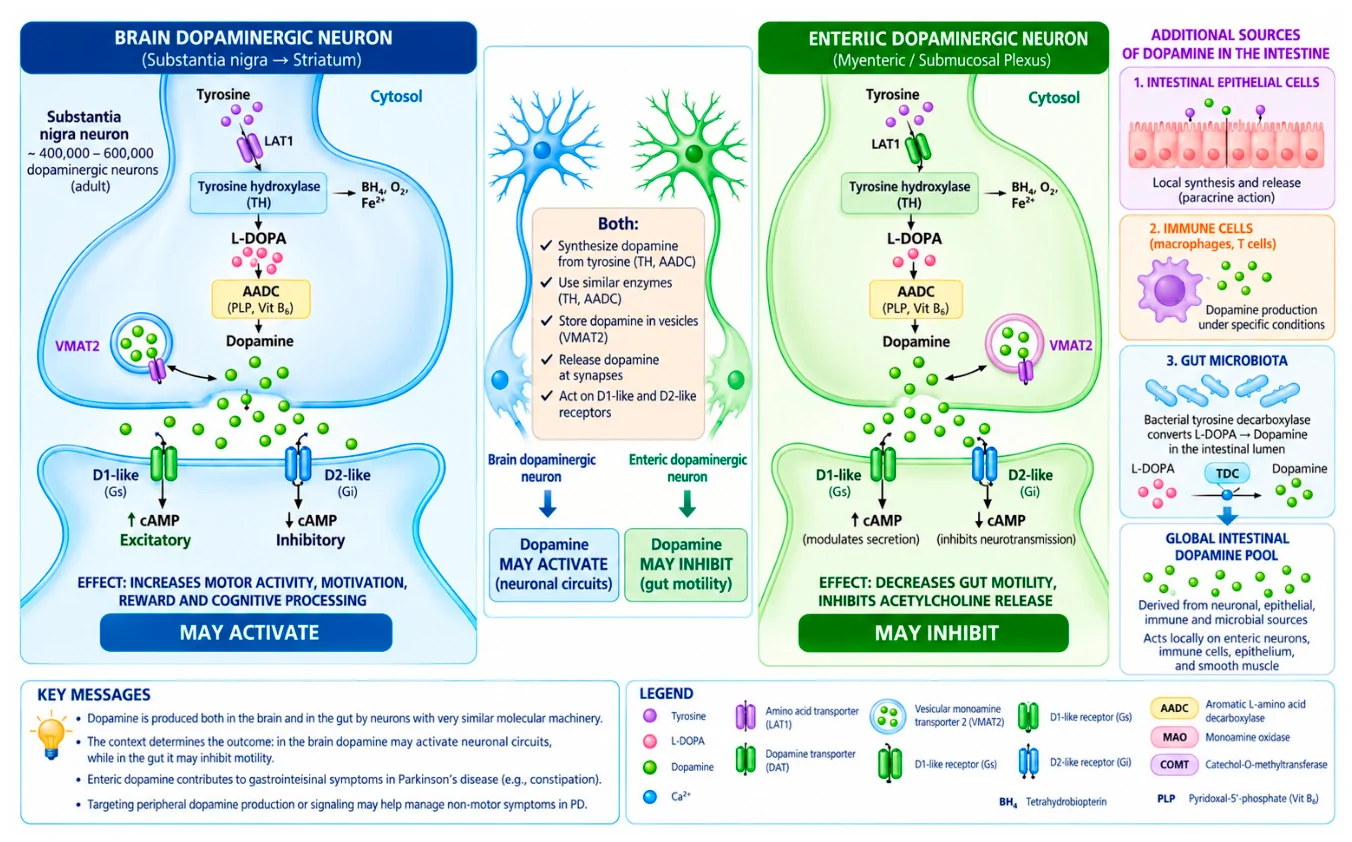
Schematic representation of dopamine synthesis, storage, and signaling in brain and enteric dopaminergic neurons. Both systems share the same molecular machinery for dopamine production and release. In the central nervous system, dopamine primarily regulates motor and cognitive functions, whereas in the enteric nervous system it modulates gastrointestinal motility and neurotransmission. Intestinal dopamine may also originate from epithelial cells, immune cells, and the gut microbiota.

Dopamine signaling is terminated mainly through reuptake by the dopamine transporter (DAT), followed by enzymatic degradation mediated by monoamine oxidase (MAO) and catechol-O-methyltransferase (COMT), ultimately generating homovanillic acid and other inactive metabolites [2] (Figure 1).

Outside the central nervous system, dopamine is synthesized in the gastrointestinal tract (enteric neurons and intestinal epithelial cells), kidneys, and adrenal medulla, where it primarily exerts local autocrine and paracrine effects rather than acting as a classical neurotransmitter [3,4]. The biosynthetic pathway of dopamine in enteric dopaminergic neurons is fundamentally identical to that in the central nervous system. However, unlike the brain, the intestine contains multiple additional sources of dopamine, including intestinal epithelial cells and gut microorganisms capable of converting luminal LD into dopamine.

Several intestinal epithelial cell types have been reported to synthesize dopamine, although the strongest evidence supports absorptive enterocytes as the principal epithelial source.

Enterocytes of the small intestine are considered the major epithelial producers of dopamine. They express Tyrosine hydroxylase, AADC/DDC, MAO and COMT. Enterocytes can: convert tyrosine to LD and then to dopamine, convert circulating or luminal LD into dopamine, release dopamine into the lamina propria and portal circulation.

The high expression of AADC in intestinal epithelial cells suggests that enterocytes are particularly efficient at converting exogenous LD into dopamine. In contrast, de novo dopamine synthesis from tyrosine appears to be more limited because tyrosine hydroxylase expression in the intestinal epithelium is relatively low and heterogeneous [5].

Consequently, intestinal dopaminergic signaling reflects the integrated contribution of neuronal, epithelial, and microbial pathways. Thus, brain dopamine is almost exclusively neuronal, whereas intestinal dopamine is neuronal, epithelial, and microbial [6,7,8].

Enteric dopaminergic neurons and dopamine receptors provide a physiological basis for local signaling. In mice, deletion of the D2 receptor accelerated gastrointestinal transit and altered colonic function, supporting an inhibitory role for this receptor in enteric regulation [9]. These findings concern endogenous enteric signaling in a controlled model. They do not demonstrate that dopamine produced by luminal bacteria reaches the same receptors, or that its contribution is quantitatively important in human constipation.

Dopamine effects depend on receptor subtype, cellular location, circuit, and exposure. A D2-mediated inhibitory enteric pathway cannot be generalized to every intestinal motor, epithelial, immune, vascular, or secretory response. Likewise, central dopamine participates in several circuits rather than producing a uniform “activating” effect. Figure 1 therefore separates luminal microbial metabolism from mucosal/enteric signaling and identifies receptor access as a missing link, instead of contrasting universally activating brain dopamine with universally inhibitory intestinal dopamine.

## 3. Physiological Role of Intestinal Dopamine

**Figure 2:**
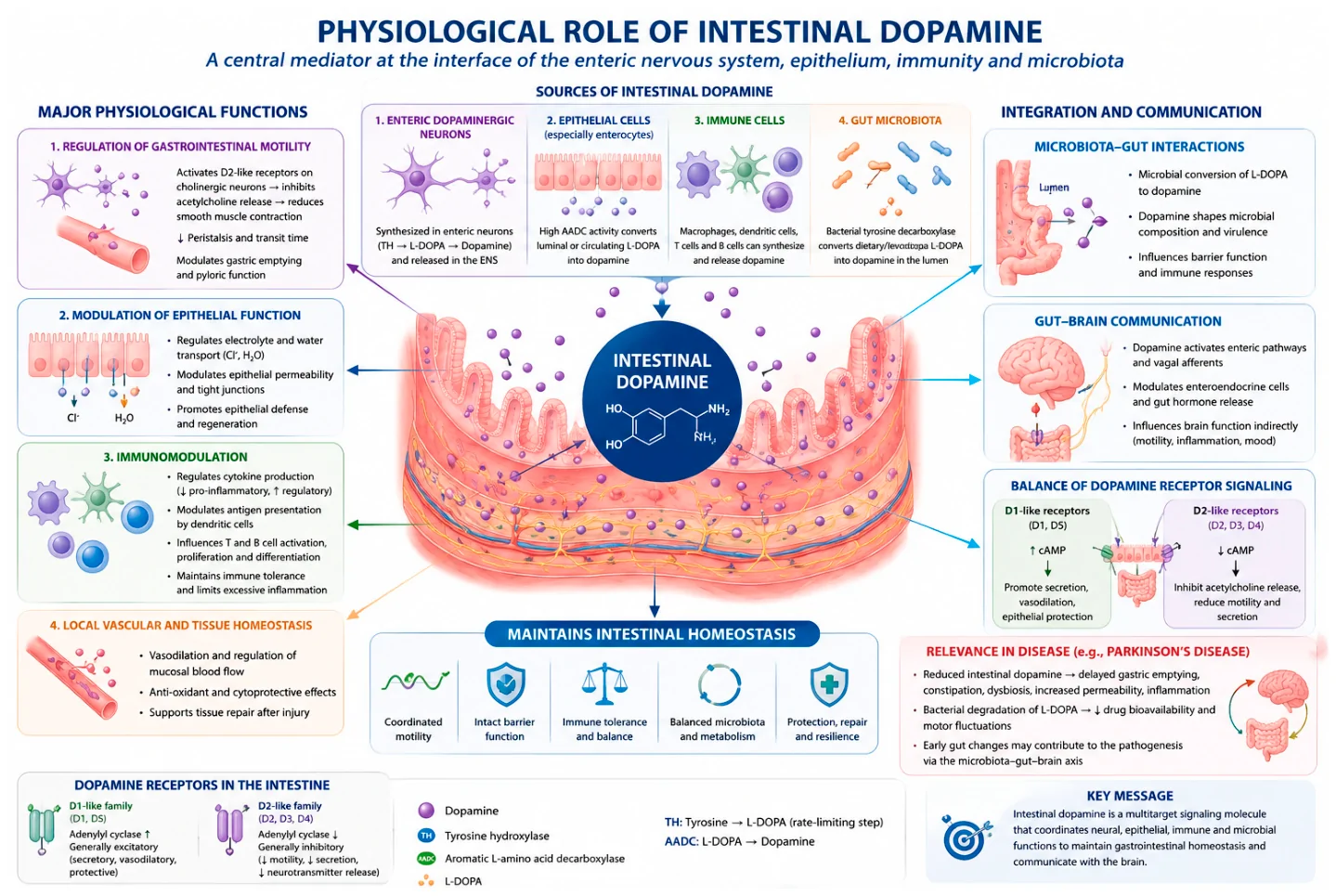
Physiological roles and sources of intestinal dopamine. Intestinal dopamine is produced by enteric dopaminergic neurons, epithelial cells, immune cells, and the gut microbiota. Through interactions with the enteric nervous system, intestinal epithelium, immune system, and microbiota, dopamine contributes to the regulation of gastrointestinal motility, epithelial barrier function, immune responses, vascular homeostasis, and gut–brain communication. The balance of D1-like and D2-like receptor signaling is essential for maintaining intestinal homeostasis and may be altered in pathological conditions such as PD.

Dopamine is an important signaling molecule within the gastrointestinal tract, where it functions as a neurotransmitter, paracrine mediator, and immunomodulatory molecule. Within the enteric system, dopamine plays a major role in regulating gastrointestinal motility. Dopamine released from enteric neurons activates predominantly D2-like receptors located on cholinergic excitatory neurons, inhibiting acetylcholine release and reducing smooth muscle contraction. Consequently, dopamine decreases intestinal peristalsis, prolongs transit time, and contributes to the physiological coordination of intestinal motor activity. In addition, dopamine modulates gastric emptying and pyloric function, thereby influencing the delivery of nutrients and orally administered drugs, including LD, to the small intestine [9,10,11] (Figure 2).

Dopamine receptors are also expressed on intestinal epithelial cells, where dopaminergic signaling influences electrolyte transport, water secretion, epithelial permeability, and mucosal barrier integrity. An additional physiological role of intestinal dopamine involves modulation of the mucosal immune system. Several intestinal immune cells, including macrophages, dendritic cells, T lymphocytes, and B lymphocytes, express dopamine receptors, while activated immune cells may also synthesize dopamine locally. Dopaminergic signaling regulates cytokine production, antigen presentation, lymphocyte proliferation, macrophage activation, and the balance between pro-inflammatory and regulatory immune responses. Consequently, dopamine contributes to maintaining immune tolerance while limiting excessive intestinal inflammation [12].

Intestinal dopamine also participates in bidirectional communication along the microbiota–gut–brain axis. Although dopamine itself does not readily cross the blood–brain barrier, it can influence neuronal activity indirectly through interactions with vagal afferents, enteroendocrine cells, and immune mediators. Conversely, the gut microbiota can modify intestinal dopamine availability by converting dietary or pharmacological LD into dopamine through bacterial tyrosine decarboxylase. This microbial metabolism has important implications for PD, as it may reduce levodopa bioavailability before intestinal absorption. The physiological actions of dopamine are mediated by five G protein-coupled receptor subtypes divided into two families. D1-like receptors (D1 and D5) stimulate adenylate cyclase and generally promote secretory and vasodilatory responses, whereas D2-like receptors (D2, D3, and D4) inhibit adenylate cyclase and predominantly suppress cholinergic neurotransmission, thereby reducing gastrointestinal motility [13,14].

## 4. What Is the Contribution of Diet to Intracerebral Dopamine Bioavailability?

**Figure 3:**
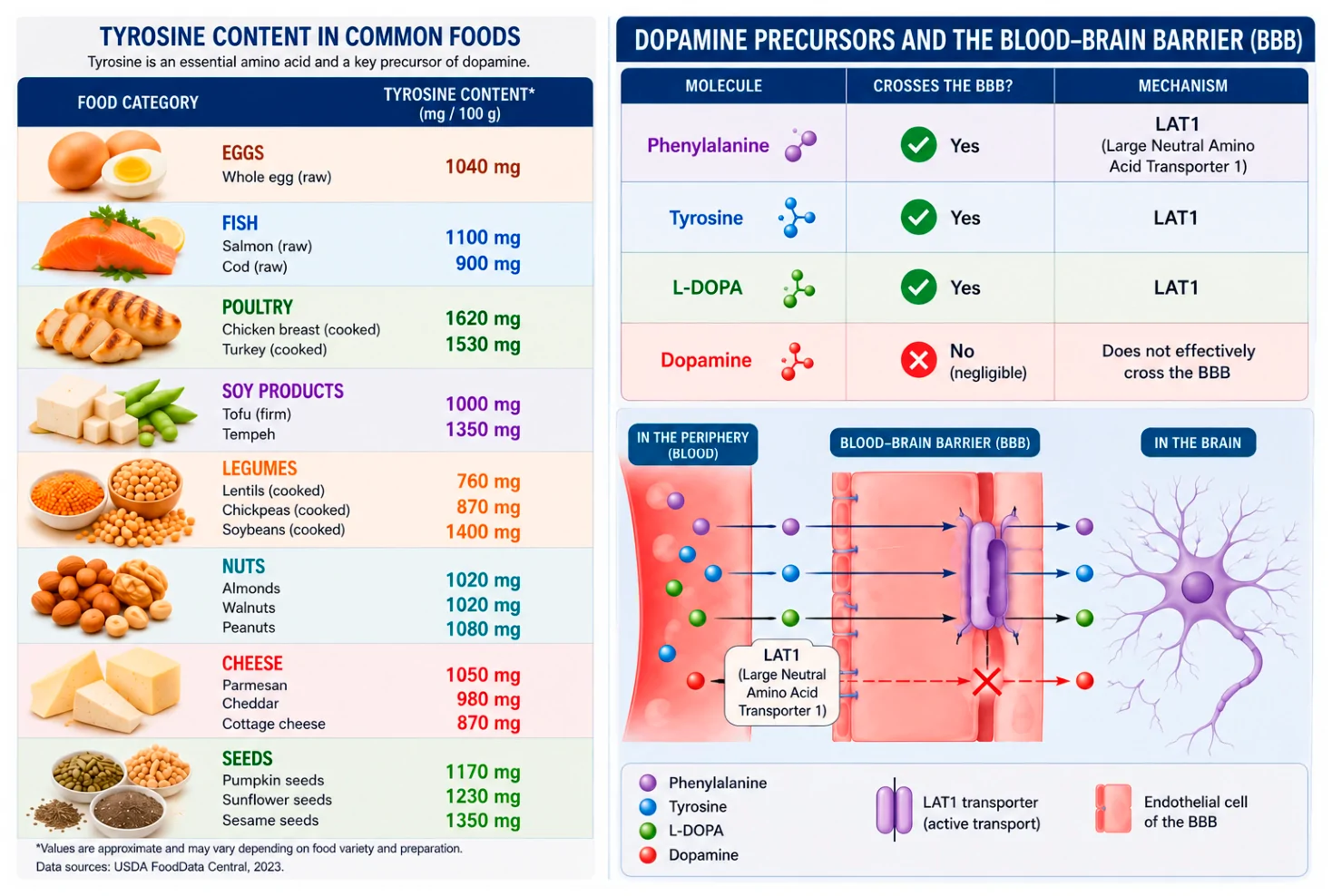
Dietary sources of tyrosine and transport of dopamine precursors across the blood–brain barrier (BBB). Tyrosine is a key dietary precursor of dopamine and is present in a variety of protein-rich foods. Phenylalanine, tyrosine, and L-DOPA can cross the BBB through the large neutral amino acid transporter 1 (LAT1), whereas dopamine itself does not effectively cross the BBB. Consequently, central dopamine synthesis depends on the brain uptake of its circulating precursors rather than on peripheral dopamine.

The amino acids phenylalanine and tyrosine, obtained from dietary proteins, are the physiological precursors of dopamine. Phenylalanine is converted into tyrosine, which is subsequently hydroxylated by tyrosine hydroxylase to form LD, the immediate precursor of dopamine. LD is then decarboxylated by AADC to generate dopamine within dopaminergic neurons. Diet contributes indirectly to dopamine synthesis by providing its amino acid precursors, tyrosine and phenylalanine. Tyrosine-rich foods include dairy products, eggs, fish, poultry, soy products, legumes, nuts, and seeds. Phenylalanine, abundant in many of the same foods, is converted into tyrosine by phenylalanine hydroxylase, thereby supporting dopamine biosynthesis [15,16] (Figure 3). Experimental studies have shown that dietary depletion of phenylalanine and tyrosine reduces brain dopamine synthesis. Consistent with this finding, positron emission tomography (PET) using [^11C]raclopride demonstrated an approximately 6% increase in D2 receptor binding following precursor depletion, reflecting reduced endogenous dopamine release [17].

These observations may indicate that adequate dietary protein intake is necessary to maintain physiological dopamine synthesis. However, increasing dietary tyrosine does not normally increase brain dopamine because dopamine synthesis is tightly regulated at multiple physiological levels. Tyrosine availability is only one of several factors controlling dopamine production, and under normal conditions it is rarely the rate-limiting step [1,18,19,20]. There are several mechanisms that limit the contribution of dietary tyrosine to the formation of intracerebral dopamine. Firstly, tyrosine transport across the blood–brain barrier is saturable. Tyrosine enters the brain through the large neutral amino acid transporter 1 (LAT1). However, LAT1 also transports several other amino acids, including phenylalanine, leucine, isoleucine, valine, tryptophan, and methionine. After a protein-rich meal, these amino acids compete for the same transporter. Therefore, simply increasing dietary tyrosine does not proportionally increase brain tyrosine concentrations. Then, tyrosine hydroxylase is the rate-limiting enzyme. Several micronutrients are also essential for optimal dopamine biosynthesis. Iron serves as a cofactor for tyrosine hydroxylase, tetrahydrobiopterin is an obligatory cofactor for tyrosine hydroxylase activity, and vitamin B6 (pyridoxal-5′-phosphate) is required for AADC-mediated conversion of LD to dopamine. Folate and vitamin B12 support one-carbon metabolism and help limit homocysteine accumulation during chronic LD therapy, whereas vitamin C may protect catecholamines from oxidative degradation. Thus, once tyrosine enters dopaminergic neurons, it must be converted into LD by tyrosine hydroxylase, which is tightly regulated by neuronal firing rate, intracellular dopamine concentrations, phosphorylation, feedback inhibition, availability of tetrahydrobiopterin, and iron availability. Even if more tyrosine is present, it does not simply produce more dopamine because enzyme activity is under strict homeostatic control. Moreover, dopamine synthesis is feedback-regulated. Dopaminergic neurons continuously monitor intracellular dopamine levels. When dopamine accumulates, tyrosine activity decreases, gene expression changes, and dopamine synthesis slows. This negative feedback prevents excessive dopamine production.

Then, we take into consideration that dopamine is stored in vesicles. Indeed, newly synthesized dopamine is rapidly transported into synaptic vesicles by VMAT2. The amount released depends primarily on neuronal electrical activity, calcium influx, and synaptic demand, rather than on dietary precursor availability. However, there are circumstances in which tyrosine supplementation may modestly enhance dopamine synthesis when there is an increment of dopamine turnover, such as prolonged cognitive work, sleep deprivation, cold exposure, military operations, and prolonged physical activity. Beyond providing biochemical substrates and cofactors, dietary patterns can influence the cerebral environment in which dopaminergic neurons function. Mediterranean-style diets, characterized by abundant consumption of vegetables, legumes, whole grains, extra-virgin olive oil, fish, fruits, and polyphenol-rich foods, are associated with reduced oxidative stress, improved endothelial function, preservation of blood–brain barrier integrity, and attenuation of neuroinflammation [21]. In addition, their high fiber content promotes the production of short-chain fatty acids (SCFAs), particularly butyrate, which enhance intestinal barrier function, promote regulatory T-cell differentiation, modulate microglial activation, and may contribute to the preservation of dopaminergic neurons through the microbiota–gut–brain axis [22].

Overall, current evidence suggests that diet influences intracerebral dopamine availability through multiple interacting mechanisms: by supplying dopamine precursors and essential enzymatic cofactors, modulating LD transport and pharmacokinetics, preserving vascular and neuronal homeostasis, and shaping the gut microbiota.

## 5. How Diet Stimulates Intestinal Dopamine

**Figure 4:**
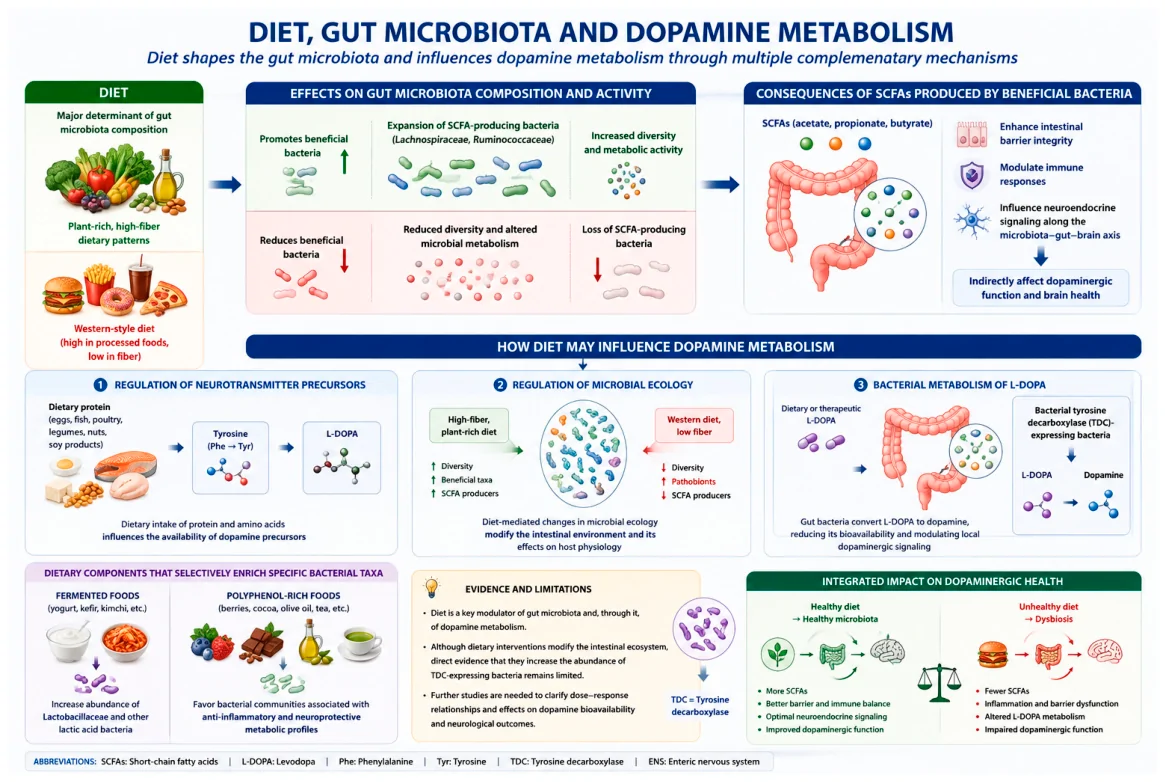
Diet, gut microbiota, and dopamine metabolism. Dietary patterns influence gut microbiota composition and activity, thereby affecting dopamine metabolism through multiple pathways. Healthy, fiber-rich diets promote beneficial bacterial communities and the production of SCFAs, whereas Western-style diets are associated with dysbiosis and reduced microbial diversity. Diet may modulate dopaminergic function by regulating the availability of dopamine precursors, shaping microbial ecology, and influencing the microbial metabolism of L-DOPA, with potential consequences for gut and brain health.

Diet is a major determinant of gut microbiota composition and may influence dopamine metabolism through multiple complementary mechanisms, including the regulation of neurotransmitter precursors, microbial ecology, and bacterial metabolism of LD (Figure 4). Diet also profoundly shapes the composition and metabolic activity of the gut microbiota. Plant-rich, high-fiber dietary patterns promote the expansion of SCFA-producing bacteria, particularly members of the *Lachnospiraceae* and *Ruminococcaceae families*, whereas Western-style diets rich in processed foods and low in fiber are associated with reduced microbial diversity and altered microbial metabolism [23]. SCFAs, including acetate, propionate, and butyrate, enhance intestinal barrier integrity, modulate immune responses, and influence neuroendocrine signaling along the microbiota–gut–brain axis, thereby indirectly affecting dopaminergic function [24]. Diet may further modulate this process: low dietary-fiber intake can alter the gut microbiota, impair mucus and epithelial barrier integrity, and increase exposure to microbial amyloids, whereas fiber-rich dietary patterns may favor SCFA-producing bacteria and support intestinal barrier homeostasis. Specific foods can selectively enrich particular bacterial taxa. Fermented foods such as yogurt, kefir, and kimchi increase the abundance of *Lactobacillaceae* and related lactic acid bacteria, whereas polyphenol-rich foods, including berries, cocoa, olive oil, and tea-favor bacterial communities associated with anti-inflammatory and neuroprotective metabolic profiles [25]. Although these dietary interventions modify the intestinal ecosystem, direct evidence that they increase the abundance of tyrosine decarboxylase (TDC)-expressing bacteria remains limited. From a pharmacological perspective, diet may also influence levodopa metabolism. *Enterococcus faecalis* and *Enterococcus faecium* express TDC, which converts LD into dopamine within the intestinal lumen before drug absorption. Subsequently, *Eggerthella lenta* can further metabolize dopamine into m-tyramine, potentially reducing LD bioavailability [26]. Consequently, dietary patterns that can reshape the abundance or metabolic activity of these bacterial taxa may contribute to interindividual variability in LD pharmacokinetics and therapeutic response. Although this hypothesis remains to be confirmed in prospective clinical studies, it provides a compelling mechanistic link between nutrition, the gut microbiome, and dopaminergic therapy in PD.

## 6. How Does Levodopa Interact with Gastrointestinal Functions, Diet, and Gastrointestinal Microbiota in PD?

### 6.1. LD and Gastrointestinal Functions

Gastrointestinal dysfunction is one of the earliest non-motor manifestations of PD and may precede the onset of motor symptoms by many years. Virtually every segment of the gastrointestinal tract can be affected, resulting in delayed gastric emptying, dysphagia, impaired intestinal transit, constipation, and malnutrition. Several mechanisms contribute to impaired LD absorption. Gastroparesis delays drug delivery to the proximal small intestine, resulting in delayed or reduced plasma concentrations and increased motor fluctuations [27,28]. In addition, LD itself may further slow gastric emptying, creating a self-perpetuating cycle of impaired drug absorption [29]. Following oral administration, LD is absorbed predominantly in the proximal small intestine through amino acid transport systems expressed by enterocytes. Experimental studies have demonstrated that intestinal uptake depends on specific transporters, such as LAT2 and TAT1, confirming that the upper small intestine is the principal site of LD absorption [30]. Despite efficient absorption, a proportion of orally administered LD remains unabsorbed and continues to transit through the gastrointestinal tract. Although direct quantification of LD in the human colon remains limited, pharmacokinetic studies and mechanistic models support the concept that residual drug can reach distal intestinal segments, particularly when small-intestinal absorption is incomplete. This phenomenon is particularly relevant in PD, where delayed gastric emptying, impaired intestinal motility, constipation, and gut dysbiosis prolong gastrointestinal transit and increase the residence time of LD within the intestinal lumen. Additional indirect evidence is provided by LD–carbidopa intestinal gel (LCIG) therapy. Although LCIG is infused into the proximal small intestine, rare mechanical complications, such as catheter migration or fistulization into the colon, demonstrate that LD formulations can be delivered to the colon under clinical conditions [31]. When LD is co-administered with a peripheral AADC inhibitor (carbidopa or benserazide), decarboxylation is markedly reduced, making COMT the predominant pathway for peripheral LD metabolism. COMT catalyzes the methylation of LD to form 3-O-methyldopa (3-OMD), an inactive metabolite. Increased formation of 3-OMD not only reduces the amount of LD available to enter the brain but may also compete with LD for transport across intestinal epithelial cells and the blood–brain barrier through large neutral amino acid transporters [32]. For this reason, COMT inhibitors, including entacapone, opicapone, and tolcapone, are widely used as adjunctive therapy in PD. By reducing peripheral LD methylation, these agents increase plasma LD concentrations, prolong its elimination half-life, decrease 3-OMD formation, and improve motor fluctuations, particularly “wearing-off” phenomena [33].

### 6.2. LD and Diet

Although COMT primarily regulates LD pharmacokinetics, adequate dopamine synthesis also depends on the availability of dietary precursors. Tyrosine and phenylalanine, supplied by protein-rich foods, provide the substrates for endogenous dopamine biosynthesis, while vitamin B6 (pyridoxal-5-phosphate) serves as an essential cofactor for AADC. Thus, dopaminergic homeostasis reflects the combined effects of pharmacological modulation of LD metabolism and nutritional support of dopamine. In patients with PD, excessive protein intake, particularly when consumed simultaneously with LD, may reduce the therapeutic efficacy of treatment. Large neutral amino acids compete with LD for transport across both the intestinal epithelium and the blood–brain barrier via the LAT1, thereby decreasing the amount of LD reaching the brain. For this reason, protein redistribution diets, in which most dietary protein is consumed during the evening meal, may improve motor fluctuations in selected patients. In PD, adherence to a Mediterranean dietary pattern, combined with appropriate timing of dietary protein intake when clinically indicated, currently represents the nutritional strategy with the strongest evidence for supporting dopaminergic function and optimizing the therapeutic efficacy of LD [34,35,36]. In this regard, we would like to mention a unique human model for understanding the relationship between genetics, metabolism, and dopaminergic function such as dopa-responsive dystonia, also known as DYT5 or Segawa disease, a rare inherited movement disorder characterized by childhood-onset dystonia, marked diurnal fluctuation of symptoms, and a dramatic and sustained response to low-dose levodopa therapy [37]. Unlike PD, DYT5 is not a neurodegenerative disorder but rather a disorder of dopamine biosynthesis. The majority of DYT5 cases result from pathogenic variants in the GCH1 gene, which encodes GTP cyclohydrolase I, the rate-limiting enzyme in the synthesis of tetrahydrobiopterin (BH4) [38]. BH4 is an essential cofactor for tyrosine hydroxylase, the enzyme responsible for converting tyrosine into LD, the immediate precursor of dopamine. Reduced BH4 availability impairs dopamine synthesis despite structurally intact nigrostriatal neurons, explaining the remarkable clinical response to LD replacement therapy [39]. Nevertheless, current evidence indicates that these nutritional influences are modest compared with the primary genetic defect, and no dietary intervention has been shown to correct the enzymatic deficiency underlying DYT5 [40,41]. Another point about LD metabolism effects and their potential correction through diet concerns the case of homocysteine, a sulfur-containing amino acid generated during methionine metabolism; its elevated plasma concentrations have been associated with an increased risk of cardiovascular disease, cognitive impairment, osteoporosis, and peripheral neuropathy. A major source of homocysteine during LD therapy is the COMT-mediated methylation of LD. COMT catalyzes the conversion of LD to 3-OMD, using S-adenosylmethionine, which is subsequently hydrolyzed to homocysteine. Consequently, prolonged LD therapy—particularly at higher doses or in the absence of COMT inhibitors—may lead to increased circulating homocysteine concentrations [42], which have been associated with cognitive decline, depression, peripheral neuropathy, and an increased risk of fractures in patients with PD [43]. Nutritional status plays a central role in homocysteine metabolism. Folate and vitamin B12 are essential for the remethylation of homocysteine to methionine, whereas vitamin B6 serves as a cofactor for the transsulfuration pathway that converts homocysteine to cysteine. Accordingly, assessment of vitamin status and supplementation when indicated represent rational strategies for reducing homocysteine concentrations during long-term levodopa treatment [44]. Monitoring homocysteine concentrations, optimizing nutritional status, and appropriately using COMT inhibitors may help reduce the long-term metabolic consequences of LD therapy and improve overall patient management [45,46].

### 6.3. LD and Gastrointestinal Microbiota

Growing evidence suggests that residual luminal LD can undergo bacterial metabolism, particularly by microorganisms expressing TDC, which convert LD into dopamine before its absorption. This microbial biotransformation may reduce LD bioavailability and contribute to interindividual variability in therapeutic response. Therefore, delayed gastrointestinal transit not only impairs LD absorption but may also increase drug–microbiota interactions, highlighting the importance of the gut microbiome as a previously underappreciated determinant of LD pharmacokinetics and clinical efficacy. Bacterial taxa capable of metabolizing LD are present in both the small intestine and the colon, although their abundance and ecological relevance differ markedly between these anatomical sites. The colon harbors the highest bacterial biomass and diversity, whereas the small intestine contains a lower but metabolically active microbial community shaped by rapid transit, bile acids, oxygen tension, antimicrobial peptides, and host immune defenses [47]. Among the microorganisms implicated in LD metabolism, *Enterococcus faecalis* and *Enterococcus faecium* provide the strongest evidence because they express TDC, an enzyme capable of converting LD into dopamine within the intestinal lumen. Certain *Lactobacillus* species also possess TDC activity, although their contribution appears to be more variable and species-dependent. Importantly, these genera are not confined to the colon. Rather than representing true bacterial “migration”, this phenomenon reflects an ecological redistribution driven by changes in the intestinal environment. Consequently, stool microbiota analyses may not accurately reflect the microbial communities of the proximal small intestine, where LD absorption occurs. This highlights the importance of investigating the small-intestinal microbiome when studying the variability of LD pharmacokinetics and therapeutic response [48]. A clinically relevant example of altered microbial distribution is small intestinal bacterial overgrowth (SIBO). In this condition, impaired intestinal motility, reduced gastric acid secretion, structural abnormalities, or altered host defenses allow bacteria that are normally more abundant in distal intestinal segments to proliferate within the small intestine. Delayed gastrointestinal transit and an increased prevalence of SIBO potentially enhance the exposure of orally administered LD to TDC-expressing bacteria before drug absorption. Also, *Helicobacter pylori* infection has also been implicated in PD. Besides its potential contribution to chronic gastrointestinal inflammation, *Helicobacter pylori* has been consistently associated with impaired LD absorption, increased motor fluctuations, and reduced therapeutic efficacy. Eradication of the infection has been shown to improve LD bioavailability and motor performance in several studies [49,50]. Consequently, a proportion of an oral dose may progress into the distal small intestine and eventually reach the colon, where absorption is considered negligible [51].

## 7. Constipation in Prodromal and Established Parkinson’s Disease: Hypothesis, Pitfalls and Predictions

### 7.1. Constipation as a Non-Motor Symptom in PD

In PD, constipation is believed to result from a multifactorial process that includes α-synuclein-related pathology within the enteric nervous system, autonomic dysfunction, impaired colonic motility, and alterations in the gut microbiota [52].

It represents one of the non-motor symptoms very common in PD that can be even more disabling than motor symptoms [53] and appear years before the clinical onset of the disease [54]. This concept is supported by neuropathological studies demonstrating *α*-synuclein pathology within the enteric nervous system of individuals with incidental Lewy body disease and patients with early PD [55]. However, non-motor symptoms are not limited to the gastrointestinal tract (constipation, gastric fullness) but also manifest elsewhere, for instance, as sleep-related behavioral disorders, hyposmia, depression, and pain. Over time, this has given rise to the “dual-hit” hypothesis, which posits the existence of two distinct clinical phenotypes of the disease, depending on two different starting points for the neurodegenerative process: “brain-first” and “body-first” [56].

### 7.2. Established Microbial Pathways

Several studies in patients with PD have reported an increased abundance of TDC bacteria converting LD and depleting SCFAs [57,58,59,60]. The two-year cohort of van Kessel et al. [60] included 67 patients with PD and 65 controls. Fecal TDC was common in both groups; abundance was not significantly different at baseline and showed only a trend toward a difference at follow-up (*p* = 0.057). The longitudinal increase was greater in PD; metagenomic studies have identified enrichment of *Enterococcus faecium*, a major TDC-expressing species, in PD [60,61]. These data establish bacterial catalytic capacity and support a drug–microbe interaction under supplied-substrate conditions.

Maini Rekdal and colleagues [62] identified a sequential pathway in which *E. faecalis* TDC forms dopamine from LD and a molybdenum-dependent enzyme in *Eggerthella lenta* converts dopamine to m-tyramine.

### 7.3. The Hypothesis of a Link Between Intestinal Dopamine and Constipation in PD

Gut microbiota should be considered an active metabolic regulator of dopamine homeostasis. The balance between dopamine-producing and dopamine-consuming bacteria, together with their enzymatic activity, may influence intestinal dopamine availability and LD pharmacokinetics, opening new perspectives for personalized treatment strategies in PD.

The excess dopamine resulting from bacterial enzymatic conversion does not cross the blood–brain barrier but remains in the intestine and can be considered one of the factors responsible for constipation in PD.

An intriguing question is whether a similar mechanism may operate before the clinical diagnosis of PD. Increasing evidence indicates that the intestinal microbiota is already altered during the prodromal phase of the disease. Microbiome studies in iRBD/prodromal PD show dysbiosis and shifts toward a PD-like microbiome [63,64,65].

**Figure 5:**
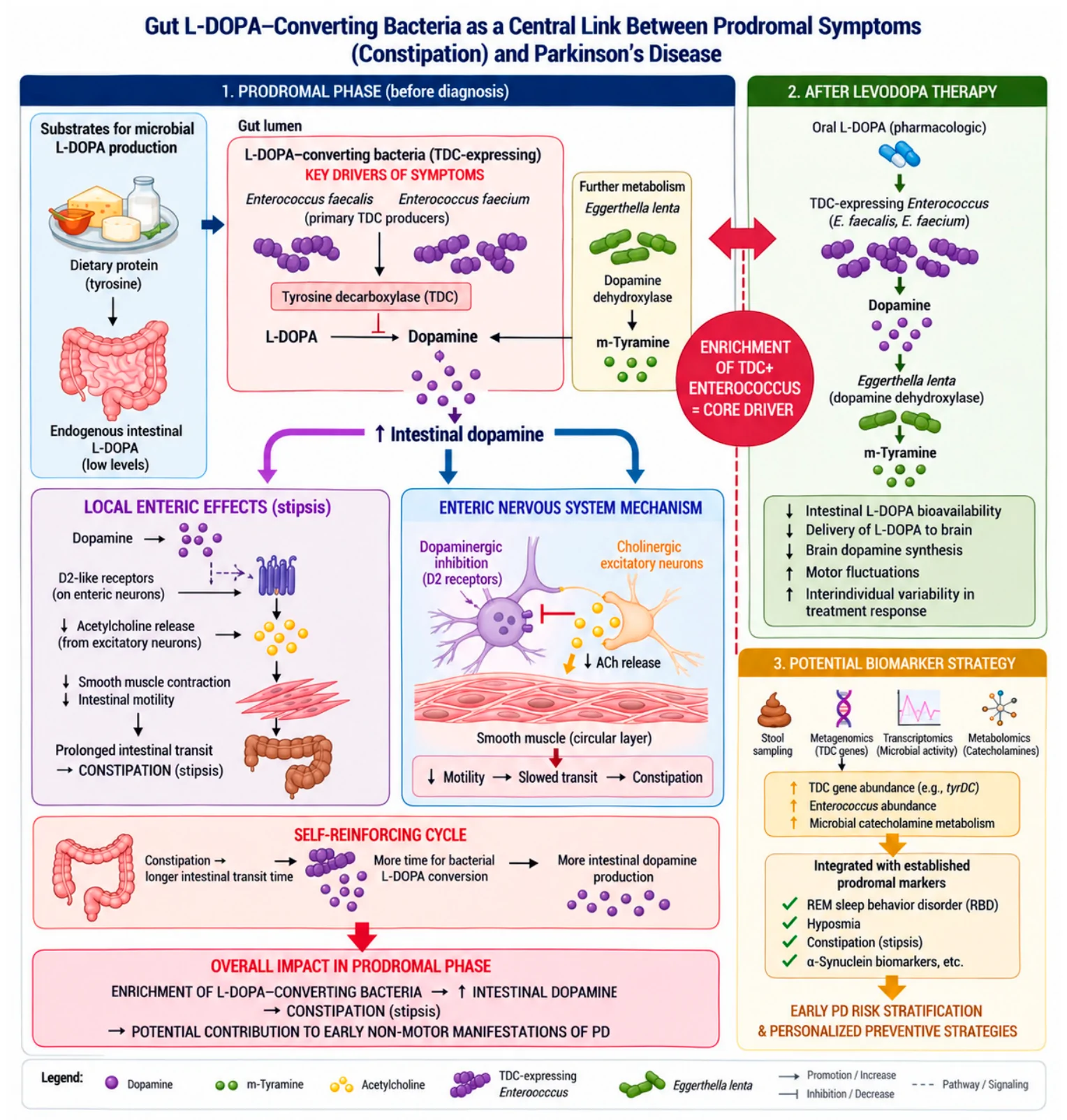
Proposed role of gut L-DOPA-converting bacteria in prodromal PD and levodopa response. Tyrosine decarboxylase (TDC)-expressing gut bacteria, particularly *Enterococcus faecalis* and *Enterococcus faecium*, can convert L-DOPA into dopamine within the intestinal lumen, potentially increasing local dopaminergic signaling. Elevated intestinal dopamine may reduce gastrointestinal motility through enteric D2-like receptor pathways, contributing to constipation, a common prodromal feature of Parkinson’s disease. During levodopa therapy, microbial L-DOPA metabolism may decrease drug bioavailability and contribute to interindividual variability in treatment response. The abundance of L-DOPA-converting bacteria and related microbial metabolites may represent potential biomarkers for early disease risk stratification and personalized therapeutic approaches.

If TDC-expressing bacterial populations are enriched during this early phase [66], they could theoretically generate increased amounts of luminal dopamine from dietary precursors [67]. Although the amount of endogenous intestinal LD available for bacterial metabolism is substantially lower than that produced during pharmacological LD therapy, even modest increases in locally generated dopamine could potentially influence enteric dopaminergic signaling [68]. Such a mechanism might contribute to delayed intestinal transit and constipation, thereby creating a self-reinforcing cycle in which prolonged luminal residence further favors microbial metabolism (Figure 5).

This hypothesis may also provide a conceptual framework linking prodromal and established PD. Before diagnosis, dietary tyrosine and endogenous LD could serve as substrates for bacterial dopamine production in susceptible individuals harboring increased numbers of TDC-expressing bacteria.

After initiation of LD therapy, the same microbial communities would metabolize pharmacological LD, reducing its intestinal availability and contributing to interindividual variability in treatment response [69]. Thus, identical microbial metabolic pathways could participate both in the prodromal phase and during chronic pharmacological treatment. If confirmed, this concept has important translational implications.

Characterization of stool microbiota, particularly the abundance of TDC-expressing bacteria and microbial genes involved in catecholamine metabolism, may provide a non-invasive biomarker for identifying individuals at increased risk of PD before the onset of motor symptoms. Integration of microbiome profiling with established prodromal markers such as REM sleep behavior disorder, hyposmia, constipation, and α-synuclein biomarkers could improve early risk stratification and facilitate the development of personalized preventive strategies.

### 7.4. Pitfalls, Suggestions and Predictions

Carbidopa or benserazide suppresses peripheral host AADC activity; this does not imply equivalent inhibition of bacterial TDC. However, we must also consider other pharmacological strategies, such as, for instance, COMT inhibitors, which modify host LD handling, while dopamine agonists may alter motility and microbial ecology [67].

Delayed gastric emptying may delay delivery of LD to its absorptive site. Doi et al. measured gastric emptying and plasma LD in patients with PD and linked impaired emptying to delayed peak concentrations [70]. Constipation, gastric emptying, and small-intestinal transit should be measured separately; one does not provide a reliable substitute for the others.

The effect of *E. lenta* must be evaluated within the context of the overall metabolic balance. Its conversion of dopamine to m-tyramine consumes dopamine and may deplete the available dopamine pool. It cannot be assumed that the physiological effect of m-tyramine mirrors D2 receptor-mediated inhibition. Strain-specific activity, substrate availability, product clearance, and the spatial overlap of the organisms involved determine the overall outcome [62].

Other microbial pathways can consume LD without producing dopamine. *Clostridium sporogenes* generates 3-(3,4-dihydroxyphenyl) propionic acid, which inhibited ileal motility in an ex vivo model and was detected in fecal samples from treated patients [26]. Commensal *bifidobacteria* can metabolize LD into 3,4-dihydroxyphenyllactic acid in vitro [71]. These findings demonstrate why a model based solely on dopamine is incomplete. Neither pathway establishes a causal link to constipation in untreated humans.

The reports on prodromal groups [63,64,65], support studying microbial changes before motor diagnosis but do not demonstrate increased TDC activity, an endogenous LD flux, or microbial dopamine-mediated constipation in those groups.

Studies should document the criteria defining the risk group and distinguish research probability estimates from an established clinical diagnosis [72]. α-Synuclein seed-amplification assays can contribute to biological characterization [73], but positivity should not be treated as proof of the proposed intestinal metabolic pathway or of its quantitative contribution to transit.

PD-related autonomic or enteric dysfunction may first slow transit, which can change microbial growth conditions and metabolite residence times. Stool consistency is strongly associated with microbiome composition and must be considered when interpreting fecal signatures [74]. Diet, hydration, physical activity, laxatives, anticholinergics, proton-pump inhibitors, antibiotics, disease severity, and treatment exposure may influence both microbial measures and gastrointestinal outcomes. An apparent TDC–constipation association can therefore arise without a causal microbial dopamine effect.

Gastrointestinal characterization should include a stool diary, Bristol stool form, complete spontaneous bowel movements, symptom severity, and Rome IV constipation criteria [75]. Objective colonic or whole-gut transit should be measured using an appropriate validated method, with evacuation disorder assessed when clinically indicated.

Diet has two different roles in this review: general support of gastrointestinal health and modification of LD exposure after treatment begins. Appropriate nutrition, hydration, and constipation management should be tailored to clinical need, while maintaining adequate protein and energy intake. Current evidence does not support dietary tyrosine restriction or supplementation as a way to manipulate the proposed prodromal pathway.

Protein amount and timing can affect LD response in selected treated patients. Carter et al. examined different protein regimens and documented differences in circulating large neutral amino acids and clinical response [76]. This supports individualized attention to diet–drug interactions, not a claim that a specific diet corrects bacterial TDC activity.

Transport competition is compartment-dependent. In the Camargo experiments, luminal leucine and arginine competed with LD uptake, whereas basolateral amino acids could stimulate efflux through exchange [30]. Consequently, meal effects cannot be reduced to a single LAT1 interaction at every barrier. Clinical interpretation requires the dose, formulation, meal timing, amino acid exposure, and gastric delivery to be specified.

A randomized trial by Barichella et al. found that fermented milk containing multiple probiotics and prebiotic fiber improved constipation in PD compared with placebo [34]. That clinical benefit does not identify TDC inhibition or reduced receptor-accessible dopamine as the mechanism. Probiotic effects are product- and strain-specific; the presence of a bacterial genus in an intervention cannot be equated with its enzyme function. Nutritional trials intended to test this hypothesis must measure conversion activity and metabolites as well as transit and clinical outcomes.

Three predictions make the proposed mechanism testable. First, in the absence of administered LD, microbial TDC activity should generate measurable dopamine from an identified luminal LD source. Second, this dopamine should become accessible to a defined receptor-bearing compartment and produce a reproducible motility response. Third, disruption of TDC or the relevant receptor pathway should diminish that response while other experimental conditions remain comparable. Failure of any required step would constrain the proposed causal chain.

The initial experiments should establish substrate sensitivity and detection limits, use stable-isotope tracing that distinguishes newly produced dopamine from background catecholamines, and include bacteria matched for background but differing in TDC function, ideally with genetic complementation. Direct addition of dopamine at the measured tissue concentrations can test sufficiency. Receptor-selective blockade or an appropriate genetic model can test dependence; bacterial viability, pH, growth, competing tyrosine, and alternative metabolites must be controlled. *E. lenta* co-culture and relevant non-dopamine pathways can then test the net product balance.

Human validation should follow successful compartmental and functional experiments. A prespecified primary prediction task is whether baseline microbial LD-conversion activity predicts subsequent worsening of objectively measured colonic transit in untreated, well-defined risk-enriched participants, conditional on baseline transit and major confounders. Incident Rome IV constipation could be a separate clinical endpoint. Later PD or other synucleinopathy conversion should be exploratory unless follow-up and sample size support that distinct endpoint; microbiome data should be tested for added value beyond existing risk markers, not substituted for them.

After participants begin LD, a separate protocol can examine standardized-dose pharmacokinetics, including Cmax, Tmax, and adequately sampled AUC. Dose, formulation, peripheral decarboxylase and COMT inhibitors, meal timing, and gastric delivery should be recorded or controlled. Associations with motor response should not be inferred from peak drug concentration alone. Training and external validation cohorts, prespecified endpoints, and assessment of calibration are required before any combined stool-based biomarker could be considered clinically useful.

Metagenomics, transcription, conversion assays, and metabolomics answer different questions (Table 1). Gene detection is not equivalent to expression; expression is not equivalent to catalytic activity; and fecal product concentration is not equivalent to tissue exposure. Integrated sampling can help resolve these steps, but adding multiple assays does not by itself establish causation or predictive value.

## 8. Conclusions

In this study, we aimed to highlight the effect of enteric dopamine on motility, which is conceptually different from that of nigrostriatal dopamine, such that the former may inhibit gastrointestinal motility while the latter may improve somatic motility. Gut bacteria are not merely passive inhabitants of the gastrointestinal tract but active participants in neurotransmitter metabolism and neurochemical signaling [77].

Bacterial metabolism of supplied LD is experimentally demonstrated and has plausible relevance to LD handling in treated PD. Its clinical magnitude and contribution to constipation remain uncertain.

TDC-expressing gut bacteria may be enriched in PD. The valorization of this knowledge may have an impact on the interpretation of constipation due to the increased release of intestinal dopamine, as well as obvious reduction of its bioavailability at the brain level. However, whether increased TDC abundance is already present during the prodromal phase of PD remains to be established.

## Figures and Tables

**Table 1:** Assays address different steps in the proposed causal chain.

Measurement	Question Addressed	Interpretive Limit
Metagenomics/TDC qPCR	Is genetic potential present?	Does not show expression or activity.
RNA/protein measurement	Is the pathway expressed?	Does not quantify conversion flux.
Controlled conversion assay	Can the sample metabolize supplied L-DOPA?	Supplied substrate may exceed endogenous exposure.
Isotope-resolved metabolomics	Which source produced each metabolite?	Sampling location and clearance remain critical.
Mucosal/tissue exposure	Does dopamine reach the target compartment?	Concentration alone does not prove receptor dependence.
Transit and receptor intervention	Is motility affected through the proposed pathway?	Confounding and off-target effects require controls.

## Data Availability

No new data were created or analyzed in this study. Data sharing is not applicable to this article.
